# The Risky Closed Economy: A Holistic, Longitudinal Approach to Studying Fear and Anxiety in Rodents

**DOI:** 10.3389/fnbeh.2020.594568

**Published:** 2020-10-23

**Authors:** Bryan P. Schuessler, Peter R. Zambetti, Kisho M. Fukuoka, Eun Joo Kim, Jeansok J. Kim

**Affiliations:** ^1^Department of Psychology, University of Washington, Seattle, WA, United States; ^2^Program in Neuroscience, University of Washington, Seattle, WA, United States

**Keywords:** fear, anxiety, decision-making, methods, ethology

## Abstract

Basic research of fear and anxiety in rodents has historically utilized a limited set of behavioral paradigms, for example, Pavlovian (classical) fear conditioning, the elevated plus-maze, or inhibitory (passive) avoidance. These traditional paradigms measure a limited selection of variables over a short duration, providing only a “snapshot” of fear and anxiety-related behavior. Overreliance on these paradigms and such behavioral snapshots ultimately lead to a narrow understanding of these complex motivational states. Here, we elaborate on the closed economy; a seldom-used paradigm that has been modified to comprehensively study fear and anxiety-related behavior and neurocircuitry in rodents. In this modified “Risky Closed Economy (RCE)” paradigm, animals live nearly uninterrupted in behavioral chambers where the need to acquire food and water and avoid threat is integrated into the task. Briefly, animals are free to acquire all of their food and water in a designated foraging zone. An unsignaled, unpredictable threat (footshock) is introduced into the foraging zone after a baseline activity and consumption period to model the risk of predation, which is then removed for a final extinction assessment. This longitudinal design, wherein data from a multitude of variables are collected automatically and continuously for 23 h/day over several weeks to months, affords a more holistic understanding of the effects of fear and anxiety on day-to-day behavior. Also, we discuss its general benefits relevant to other topics in neuroscience research, its limitations, and present data demonstrating for the first time The Risky Closed Economy’s viability in mice.

## Introduction

Neuroscience techniques are becoming exponentially more sophisticated, allowing researchers to measure and manipulate the brain at larger scales with more precision and specificity. However, in rodent fear and anxiety research, what appears to remain static is the use of a limited set of behavioral paradigms in which these new technologies are being employed (Mobbs and Kim, 2015; Kim and Jung, 2018). Examples include Pavlovian (classical) fear conditioning and the freezing response (Fanselow, 1980), the elevated plus-maze and time spent in open arms (Pellow et al., 1985), and inhibitory (passive) avoidance and latency to enter a shock-associated dark compartment (Venable and Kelly, 1990; Deakin and Graeff, 1991). While such paradigms have yielded invaluable insights, they are usually short (typically minutes) and measure a narrow range of behaviors, in effect providing only a “snapshot” of a given phenomenon (Pellman and Kim, 2016). For example, this brief sampling of behavior excludes temporal aspects of fear and anxiety, including how fear and anxiety-related behavior vary over time, as well as how fear and anxiety affect circadian/infradian rhythms and long-term, risky decision-making.

On one hand, the continued use of these customary paradigms allows researchers to focus on thoroughly mapping and characterizing the neurocircuitry of a small set of well-known, predictable behaviors. On the other hand, their overreliance leads to gaps in knowledge regarding the behaviors and neurophysiology associated with fear and anxiety in rodents and thus a restriction on translational potential (Pellman and Kim, 2016). What is needed also are paradigms that can more comprehensively model in rodents the complexities of normative fear and anxiety. One such paradigm that provides the foundation for a more holistic approach to studying rodent behavior is the “closed economy”; a paradigm in which animals obtain their daily food exclusively through operant responding and typically live in the operant chambers themselves (Collier et al., 1972; Collier, 1983). By introducing an aversive component to the closed economy—namely, context-dependent, unpredictable footshock lasting several weeks (Fanselow et al., 1988; Helmstetter and Fanselow, 1993)—a unique, naturalistic chronic approach/avoid conflict is engendered; “The Risky Closed Economy (RCE)”. With the addition of modern animal tracking and automation technologies (Kim et al., 2014), the RCE allows for fear and anxiety-related behavior to be expansively studied.

## Historical Origins

**Figure 1:**
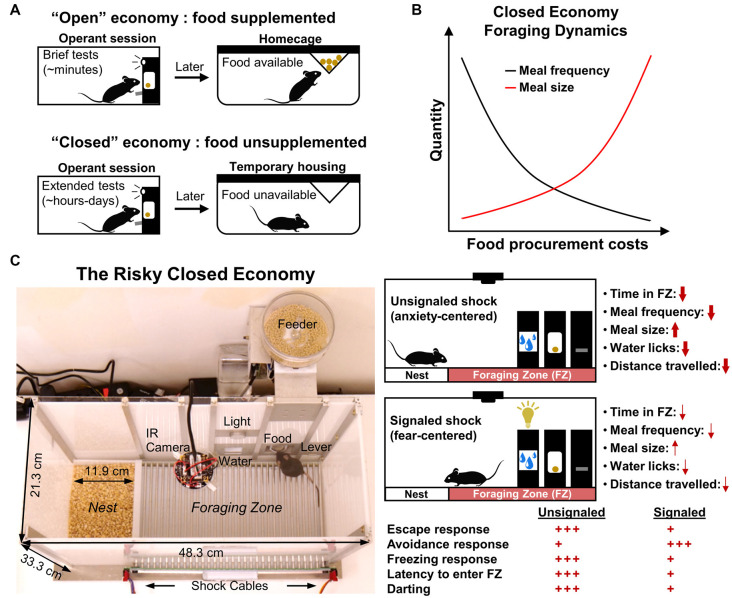
Traditional closed economy concepts and The Risky Closed Economy (RCE). **(A)** In “closed economies,” food and/or water is not supplemented after testing; animals must earn all of their food and/or water during the testing session, reflecting naturalistic conditions. Closed economy experiments also typically utilize long testing periods. This contrasts with “open economies” where food is supplemented (e.g., food restriction) after brief tests. **(B)** The use of chained schedules of reinforcement in closed economy experiments (e.g., fixed ratio-continuous reinforcement) allows for discrete eating bouts (“meals”) and the number of pellets per eating bout (“meal size”) to be measured. Under optimal experimental parameters, closed economy animals characteristically decrease the frequency of meals and increase meal size in response to increasing food procurement costs. **(C)** In the RCE, pseudo-random footshock is integrated into a longitudinal closed economy framework as a means to model naturalistic risky foraging with predation threat. Use of an unsignaled (no cue) or signaled (cued) shock allows one to investigate the effects of diffuse, anxiety-evoking, or imminent, fear-evoking threat, respectively, on circadian and infradian behavior. Red arrow thickness and quantity of red plus signs represent the impact of shock condition on the listed behavioral variables under optimal experimental parameters (i.e., from rat studies mentioned in the text). Darting: sudden activity bursts exceeding 23.6 cm/s.

In economic terms, a closed economy refers to an ideal state in which daily consumption is the result of the equilibrium of supply and demand. As it applies to animal research, a closed economy refers to a scenario in which the animal’s consumption of food (demand) results solely from its interaction with schedules of reinforcement (supply); that is, the animal controls its total food intake *via* operant responding without experimenter food supplementation (Hursh, 1980). This contrasts with an “open economy,” where food is supplemented outside of the operant session and thus behavior within the session is independent of total daily consumption of the reinforcer (Hursh, 1980, 1984). Also, characteristic of animal closed economy experiments are long measurement sessions—typically 23 h per day over several days—sufficient within-session reward densities suitable for survival and deprivation levels that are determined by the animal’s within-session food intake (Timberlake and Peden, 1987; Posadas-Sanchez and Killeen, 2005; Figure 1A). Collier et al. (1972) were the first to characterize rats’ foraging behavior as a function of effort in a closed economic system and showed that male rats exhibit robust operant responding for food pellets at unusually high reinforcement schedules when daily food consumption was made entirely contingent on the animals’ behavior, as would be the case in the animals’ natural environment. They additionally demonstrated that rats tend to decrease eating bouts while increasing pellets obtained per eating bout in response to increasing schedule demands, following optimal foraging strategy, which postulates that animals strive to maximize caloric intake while minimizing energy and time costs (MacArthur and Pianka, 1966; Figure 1B). Importantly, these results contrast with studies that show that animals are far less willing to engage similar schedules of reinforcement when food is supplemented outside the testing session, emphasizing the notion that the animal’s total reinforcer economy and their experience outside of the testing session are crucial determinants of operant behavior within the testing session (Hursh, 1978, 1980; Kearns, 2019; but see Timberlake and Peden, 1987). Since these original studies, much research has been dedicated to exploring the influences of open economies vs. closed economies on operant behavior in a variety of species (for review see Posadas-Sanchez and Killeen, 2005; Kearns, 2019).

## Naturalistic Qualities

The closed economy approximates a naturalistic foraging scenario. As mentioned above, the animal alone dictates its daily nutrition but must exert effort to obtain sustenance. This effort component arises from a chained schedule of reinforcement, which is used to model the time/energy costs associated with initially procuring the food item and the subsequent energy/time costs associated with manipulating and consuming the food item while foraging (Collier, 1983). In completing the procurement phase of the schedule (first chain component; e.g., fixed-ratio 50, FR50), the animal initiates a “meal” and then transitions into the consumption phase (second chain component; e.g., continuous reinforcement, CRF), where it may obtain food as long as it continues to emit operant responses. A meal ends when the animal fails to respond after a set amount of time in the consumption phase, which resets the schedule and ends the meal. The amount of food obtained during a meal is referred to as the “meal size.” Therefore, in response to shifting foraging constraints, the animal can choose to alter its foraging strategy by changing parameters such as daily meal frequency, meal size, and response rate (Collier, 1983).

The closed economy is further made naturalistic when a risk component is added to the foraging experience. In nature, animals must often leave the safety of their nests to forage in potentially dangerous locations (Lima and Valone, 1986). Fanselow et al. (1988) housed female rats in operant chambers that included a safe “Nest Zone,” comprised of sawdust bedding and a water bottle, and a risky “Foraging Zone,” which contained a shock grid floor and the operant lever/food port. After a baseline foraging and activity period, unsignaled but escapable footshocks were administered in the Foraging Zone for several weeks to model an additional cost associated with naturalistic foraging: predation (Krebs, 1980). These unpredictable shocks were then terminated for a “Post-Shock” (“Extinction”) assessment. Rats responded to this persistent threat by decreasing meal frequency but compensated caloric intake by increasing meal size (Figure 1B). This strategy, coupled with a strong avoidance of the risky Foraging Zone during the “Shock” phase, allowed animals to continue to gain weight and minimized the amount of daily footshock received. The results of this study and future studies expanding on this paradigm (Helmstetter and Fanselow, 1993; Kim et al., 2014; Pellman et al., 2015, 2017) support the ethological theory that animals integrate the risk of predation in their daily foraging and activity decisions (Lima and Dill, 1990). Note that footshock is not intended to represent predatory encounter *per se*, but is used to broadly model the risk of predation while foraging. The incorporation of risk into the closed economy framework and longitudinal design form the basis of the RCE and enhance the paradigm to achieve greater ethological relevance (Figure 1C). In the RCE, the need to acquire food and water while avoiding unpredictable threat is integrated into the animals’ lives—an ubiquitous scenario in nature (Mobbs and Kim, 2015). The naturalistic qualities and longitudinal design of the RCE provide unique benefits compared to traditional methods (Table 1) as discussed in the proceeding sections.

**Table 1:** Advantages of the Risky Closed Economy (RCE) paradigm relative to traditional fear and anxiety paradigms, such as Pavlovian (classical) fear conditioning, the elevated plus-maze, and inhibitory (passive) avoidance paradigms.

The Risky Closed Economy	Traditional fear/Anxiety paradigms
Longitudinal; 23 h/day data collection for several weeks.	Brief tests offering only “snapshots” of behavior.
A multitude of behavioral variables (holistic approach).	Few behavioral variables (hyper-focused approach).
Naturalistic. A risky-foraging scenario requiring effort and decision-making; the need to acquire food and water while avoiding unpredictable threats is integrated into the animals’ lives. The ethologically-relevant, goal-oriented (purposive) task facilitates the interpretation of behavior.	Less naturalistic. Food and water are provided and/or restricted by the experimenter. Small chambers and short test duration constrain the animals’ behavioral repertoire.
Minimal experimenter interaction.	Increased experimenter interaction (handling, feeding, and frequent transport).

## Utility in Fear and Anxiety Research

When shocks are delivered unpredictably, the Shock phase of the RCE most suitably evokes anxiety (Fanselow et al., 1988; Helmstetter and Fanselow, 1993; Kim et al., 2014; Pellman et al., 2015, 2017; Figure 1C, top right). According to Predatory Imminence theory, which proposes that organisms’ momentary perception of predation risk determines their defensive behavioral topography (Fanselow and Lester, 1988), the subtle changes in meal patterns and avoidance resulting from these shock parameters in the RCE resemble “pre-encounter” defensive reactions to threat (Fanselow et al., 1988; Helmstetter and Fanselow, 1993). The pre-encounter phase is defined as a situation in which there is a possibility of harm but is low in the probability or distant and is accompanied by anxiety-like reactions such as avoidance, risk assessment, and vigilance meant to decrease the chances of encountering danger (Perusini and Fanselow, 2015). The diffuse and unpredictable nature of shock using these parameters and the observed defensive behavior also aligns well with the “sustained fear” concept of anxiety, where the defensive behavior maintains long after the aversive stimulus is removed (Davis et al., 2010).

In a general sense, this paradigm shares features with traditional punishment-based approach-avoid conflict paradigms used to screen anxiolytics, such as the Vogel Conflict Test (Vogel et al., 1971) and Geller-Seifter test (Geller et al., 1962). It therefore may be useful for studies investigating the longitudinal effects of anti-anxiety medications on factors such as avoidance, decision-making, feeding behavior, and sleep/wake cycles. The RCE also shares qualities with the platform-mediated avoidance paradigm (Bravo-Rivera et al., 2014) where rats are trained to lever press for food then receive tone-shock pairings, such that when the tone is presented animals escape the shock-grid to a nearby platform. Likewise, when a signaled shock is utilized in the RCE, the paradigm contains elements found in condition suppression tasks, where the presence of a cue paired with shock terminates lever-pressing behavior (Estes and Skinner, 1941). However, these acute behavioral paradigms do not afford a comprehensive picture of the effects of threat on day-to-day behavior, measure fewer variables over shorter periods, and involve food restriction/supplementation (open economy) which can affect operant behavior (Hursh, 1980; Table 1).

The use of a discrete cue preceding shock in the Foraging Zone, such as a light, may be used to invoke fear (Figure 1C, bottom right). Fear is typically conceptualized as a defensive state resulting from the imminent, predictable threat with behaviors and neural substrates dissociable from that of anxiety (Davis, 1998; Perusini and Fanselow, 2015; Robinson et al., 2019). Indeed, when threat cues are utilized within the RCE, the foraging and activity level changes seen in the standard unsignaled shock condition are near absent, as active avoidance takes precedence over passive avoidance responses (Pellman et al., 2015). The paradigm may therefore be used to study continuously the development of Pavlovian instrumental transfer under more naturalistic conditions, or in the case of consecutive unsignaled to signaled Shock phases, whether a neural manipulation disrupts both contextual and/or discrete cue learning.

The RCE affords researchers a means to study facets of fear and anxiety-related behavior typically not feasible in the predominant paradigms mentioned above. One facet includes the spatiotemporal dimension of fear and anxiety. In segmenting the apparatus into distinct “risky” vs. “safe” zones and by continuously measuring the animal’s behavior for extended periods, one can investigate how context and prolonged exposure to aversive stimuli interact to shape the animal’s day-to-day behavior. For example, it has been shown that threat associated with the Foraging Zone during the dark portion of the dark/light cycle can change rats’ foraging and overall activity to occur primarily during the light portion of the dark/light cycle, essentially reversing the animals’ typical circadian activity patterns (Pellman et al., 2015). The paradigm may therefore be of use in research centered on fear and anxiety’s disruptive effects on circadian rhythm, which is known to be dysregulated in human anxiety and mood disorders (Amir and Stewart, 1998; Roybal et al., 2007; American Psychiatric Association, 2013; Walker et al., 2020). Given that anxiety disorders emerge early on in life (Pine, 2007), the RCE could also be used in developmental research investigating the impact of chronic, unpredictable threat or cyclic threat on anxiety, fear, and decision-making behavior in different age groups, or as an initial screening for individual differences in trait anxiety. Finally, due to the delineation of safe vs. risk zones, risk assessment behaviors such as a stretched, attentive posture toward the source of threat (Blanchard et al., 1990; Choi and Kim, 2010) may also be examined in addition to standard freezing and avoidance metrics (Figure 1C).

The naturalistic qualities of the RCE facilitate the assessment of fear and anxiety in decision-making (Mobbs et al., 2018), a form of executive functioning (Robinson et al., 2013). Aside from the aforementioned changes in meal patterns and avoidance, other forms of decision-making under risk can be examined with the creative use of the operant devices and reinforcement contingencies used to simulate the work component of foraging. Kim et al. (2014) incorporated two operant levers; one located close to the safe Nest Zone and another located on the same wall of the apparatus but at the distal end of the Foraging Zone. Results indicated that amygdala lesioned rats with an initial preference for the farther lever failed to switch to the closer, safer lever during the Shock phase unlike sham lesioned controls. In this study, the authors varied lever distance to probe the animals’ distance gradient of fear and its influence on appetitive behavior, but similar methods can be used to study a variety of other cognitive processes. For example, one could utilize two levers equidistant from the nest area that offer either high reward at low probability or low reward at high probability, respectively, to examine how the risk of shock influences impulsivity under closed economy conditions (Green and Myerson, 2004). In a broad sense, the RCE can also be used to examine in rodents human behavioral economic principles, such as those outlined in Kahneman and Tversky’s Prospect Theory (1979), which proposes that decisions under risk are subject to the influence of past outcomes and cognitive biases that promote or inhibit risk-taking behavior. For example, one can examine how different levels of anxiety/fear interact with different levels of weight loss over time to promote risky foraging or avoidance.

## Viability in Mice

**Figure 2:**
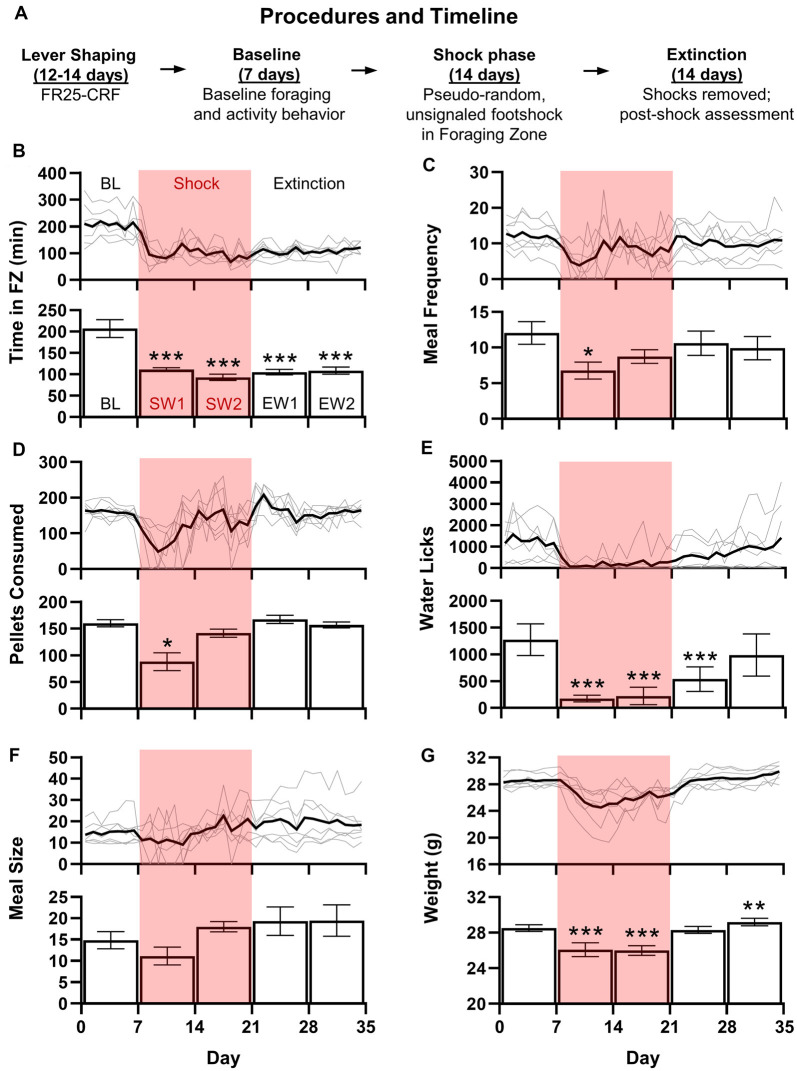
Mice are a viable alternative to rats in RCE experiments. **(A)** Upon arrival, animals were immediately housed in RCE Chambers distributed between two rooms (four chambers/room). Mice were then shaped to lever press for food at a fixed ratio 25-continuous chained schedule of reinforcement (FR25-CRF), wherein each press beginning at FR25 results in one pellet/press. The schedule resets after 1 min of lever inactivity. A “meal” occurs when the FR threshold is met and continues until the scheduled reset. Shaping begins at FR1 and doubles every 2 days until FR25 (except FR16 transitions to FR25). Baseline foraging and activity level assessment follow until 7 days of stable behavior are obtained. Unsignaled, pseudo-random (~2/h) footshocks (0.5 mA, 10 s or until escape to Nest Zone; 48 shocks/day max) are introduced in the Foraging Zone for a 2-week Shock phase. Finally, shocks are terminated for a 2-week Extinction phase. **(B–G)** shows the daily average (black line) with individual mouse data (gray lines; top) and weekly average ± SEM (bottom) for daily total time spent (minutes) in the Foraging Zone **(B)**, meal frequency (or the number of meals/day; **C**), food pellets consumed **(D)**, water lick meter beam breaks **(E)**, meal size (food pellets/meal; **F**), and animal weight (grams; **G**) of adult male C57BL/6 mice (3 months old upon arrival; *N* = 7) across Baseline (BL), Shock (S) and Extinction (E) phases (*p < 0.05, **p < 0.01, ***p < 0.0001 vs. Baseline).

Studying fear and anxiety using the RCE is also feasible in mice, thus opening the door for use of transgenic mouse models. Figure 1C (left) depicts the mouse-adapted RCE. The animal position is tracked *via* a mounted infrared camera connected to a central computer running ANY-maze tracking software (RRID: SCR_014289). The software also quantifies lever presses/licks, triggers the pellet dispenser (ENV-310W, ENV-251M, ENV-203M-45; Med Associates, Fairfax, VT, USA) to deliver 20 mg dustless precision food pellets (F0163; Bio-Serv, Flemington, NJ, USA), and triggers the precision animal shocker to deliver shocks to Forging Zone steel grid floor (H13–15, H10–11M–XX–SF; Coulbourn, Holliston, MA, USA). One central computer with ANY-maze software and interface accommodates four chambers. Mice (*N* = 7) proceeded through Lever Shaping, Baseline, Shock, and Extinction phases as outlined in Figure 2A. Figures 2B–G show adult (3 months) male C57BL/6 mouse (IMSR Cat# CRL_27, RRID:IMSR_CRL:27) data. Custom Python scripts were used for data aggregation and the formation of custom variables. Parametric data were analyzed with one factor repeated measure ANOVAs followed by Bonferroni-corrected Dunnett’s *post hoc* comparisons in Prism (GraphPad Prism, RRID:SCR_002798). Greenhouse-Geisser corrected degrees of freedom were used when the sphericity assumption was violated (Mauchly’s test). Non-parametric data were analyzed with rank-based repeated measure ANOVAs followed by Bonferroni-corrected multiple comparisons using the R package nparLD (Noguchi et al., 2012). All statistical tests were performed with an alpha level set to 0.05.

The introduction of unsignaled, pseudo-random (~2/h) footshocks (0.5 mA, 10 s or until escape to Nest Zone; 48 shocks/day max) significantly reduced time spent per day (*F*_(4,24)_ = 17.89, *p* < 0.0001; Figure 2B) in the Foraging Zone during both Shock and Extinction phases relative to Baseline (*p*’s < 0.0001). Footshock also reduced the daily meal frequency (*F*_(2.425)_ = 2.802, *p* < 0.05; Figure 2C) and food pellets consumed (*F*_(1.521,9.125)_ = 11.11, *p* < 0.01; Figure 2D) during the first week of shocks (*p*’s < 0.05), which recovered to Baseline levels by week 2 of the Shock phase. Footshock significantly decreased the number of water licks per day (*F*_(2.557)_ = 11.191, *p* < 0.0001; Figure 2E) throughout the Shock phase and first week of Extinction (*p*’s < 0.0001) but was not significantly different from Baseline by Extinction week 2. There were no significant changes in meal size (*F*_(1.556, 9.337)_ = 2.744, *p* = 0.1224; Figure 2F). Finally, footshock depressed weight (*F*_(1.884)_ = 33.228, *p* < 0.0001; Figure 2G) during the Shock phase, which returned to baseline levels during the first week of Extinction and exceeded Baseline levels by Extinction week 2 (*p*’s < 0.01). Ultimately, mice behaved similarly to adult female Long-Evans rats in the RCE that experienced comparable shock frequency (Pellman et al., 2017); mice did not increase the number of pellets consumed per eating bout to offset the decreased eating bouts per day during the Shock phase, lost weight, and did not extinguish avoidance of the Foraging Zone after the shock was removed. The shock frequency selected based on the performance of male rats in previous studies conducted in our laboratory proved too aversive for our male mice and likely prevented them from displaying the abovementioned meal alterations (Helmstetter and Fanselow, 1993). Given that footshock strongly inhibited foraging, the reduction in aversion resulting from avoidance likely negatively reinforced the behavior to a degree that prevented the extinction of avoidance when the shock was terminated (Mowrer, 1939). Thus, the data presented here may not reflect an optimized version of the task; future experiments adjusting lever contingencies and/or shock intensity and frequency are warranted.

## Discussion

The RCE’s unique longitudinal design and ethological qualities serve to expand both the animal’s behavioral repertoire and what can be measured in a controlled laboratory setting. Data obtained from such goal-oriented (Tolman, 1932), “big picture” analyses can be used to further refine our understanding of rodent fear and anxiety and subsequently aid in mapping their behavior onto human behavior to enhance translational relevance. Similarly, by studying neural mechanisms under more ethological conditions, a more accurate understanding of how these mechanisms naturally operate may be achieved. Understanding how these mechanisms operate in situations they likely evolved to handle can pave the way for understanding how they malfunction in mental illness. With recent advances in tracking software, reversible, time-specific neural manipulation techniques, wireless recording/optogenetics technologies, and increased feasibility of big data analysis, the RCE has the potential to generate a wealth of knowledge regarding the neural circuitry of fear and anxiety-related behavior.

The RCE concept and apparatus offer benefit applicable to behavioral testing in general. Our design (Figure 1C) allows for automated acquisition and scoring of behavioral variables, reducing experimenter biases that negatively impact a study’s validity and reproducibility (Barber and Silver, 1968). This automation further assists in the RCE’s “hands-off” approach that minimizes experimenter-subject interaction. For example, in our procedure, animals are removed from their chambers for only 1 h/day for apparatus maintenance and health checks; animals are left undisturbed the remaining 23 h of the day. Importantly, limiting experimenter-subject interaction reduces potential stress on the animals (Hurst and West, 2010; Sorge et al., 2014), which improves overall animal wellbeing and reduces study variability. Unlike other relatively chronic tasks in rodents, such as “touchscreen” paradigms (Delotterie et al., 2014), no training is required to perform the task, as animals are autoshaped to procure food and water and no food deprivation is imposed. Lastly, because the animal’s home cage is the testing apparatus itself and behavior are measured nearly continuously for extended periods, post-surgery changes in baseline behavior can be screened before testing animals under new experimental conditions. This helps clarify test results and interpretations and is especially relevant to research incorporating irreversible neural manipulations, such as electrolytic, chemical, or genetic lesions.

Although we encourage the implementation of the RCE in neuroscience research, we acknowledge that the paradigm has limitations that make it impractical for certain research projects. The most obvious is that by design, RCE experiments take a substantial amount of time to complete (Figure 2A). Thus, for those seeking to adopt the paradigm, multiple chambers should be constructed (at least 8). We also recognize that in its current configuration (Figure 1C), the RCE introduces social isolation as a factor. This can be partially ameliorated by having clear, perforated acrylic chamber walls where animals can both see and smell each other and by group-housing animals during the daily 1-h removal period. The longitudinal aspect and enclosed chamber also make the use of certain tools, like tethered optogenetics and electrophysiological recording, challenging. However, careful planning and wireless alternatives can overcome this obstacle to provide future studies a powerful means by which to study the neural mechanisms of complex behaviors over time. For example, pharmacological and/or chemogenetic tools such as Designer Receptors Exclusively Activated by Designer Drugs (Armbruster et al., 2007) are suitable for use in RCE experiments; treatment may be given on alternating days (A-B-A-B design) either manually through injections/infusions or remotely with programmable minipumps (see iPRECIO Programmable Infusion Pump; ALZET Osmotic Pumps, Cupertino, CA, USA). Finally, given the viability of mice in the RCE, transgenic strains may also be taken advantage of. In sum, the RCE provides both unique advantages and opportunities relative to more traditional fear and anxiety paradigms and general benefits applicable to other subfields within neuroscience research.

## Data Availability Statement

All datasets presented in this study are included in the article.

## Ethics Statement

The animal study was reviewed and approved by the University of Washington Institutional Animal Care and Use Committee.

## Author Contributions

BS conducted the research and analyzed the data. BS and KK designed and constructed the behavioral apparatus. BS, KK, EK, PZ, and JK wrote and revised the manuscript. All authors contributed to the article and approved the submitted version.

## Conflict of Interest

The authors declare that the research was conducted in the absence of any commercial or financial relationships that could be construed as a potential conflict of interest.
