# Corrigendum: The Risky Closed Economy: A Holistic, Longitudinal Approach to Studying Fear and Anxiety in Rodents

**DOI:** 10.3389/fnbeh.2021.684179

**Published:** 2021-04-09

**Authors:** Bryan P. Schuessler, Peter R. Zambetti, Kisho M. Fukuoka, Eun Joo Kim, Jeansok J. Kim

**Affiliations:** ^1^Department of Psychology, University of Washington, Seattle, WA, United States; ^2^Program in Neuroscience, University of Washington, Seattle, WA, United States

**Keywords:** fear, anxiety, decision-making, methods, ethology

An author name was incorrectly spelled as **Kisho M. Kukuoka**. The correct spelling is **Kisho M. Fukuoka**.

The authors apologize for this error and state that this does not change the scientific conclusions of the article in any way. The original article has been updated.

